# Formaldehyde formation in the glycine cleavage system and its use for an aldolase-based biosynthesis of 1,3-propanediol

**DOI:** 10.1186/s13036-020-00237-2

**Published:** 2020-05-14

**Authors:** Yingying Xu, Hao Meng, Jie Ren, An-Ping Zeng

**Affiliations:** 1 Beijing Advanced Innovation Center for Soft Matter Science and Engineering, Beijing University of Chemical Technology, North Third Ring Road 15, Chaoyang District, Beijing, 100029 China; 2 State Key Laboratory for Biology of Plant Diseases and Insect Pests/Key Laboratory of Control of Biological Hazard Factors (Plant Origin) for Agri-product Quality and Safety, Ministry of Agriculture, Institute of Plant Protection, Chinese Academy of Agricultural Sciences, Beijing, 100081 China; 3 Institute of Bioprocess and Biosystems Engineering, Hamburg University of Technology, Denickestrasse 15, D-21073 Hamburg, Germany

**Keywords:** Glycine cleavage system, Formaldehyde, 5,10-CH_2_-THF, 1,3- propanediol

## Abstract

Glycine cleavage system (GCS) occupies a key position in one-carbon (C1) metabolic pathway and receives great attention for the use of C1 carbons like formate and CO_2_ via synthetic biology. In this work, we demonstrate that formaldehyde exists as a substantial byproduct of the GCS reaction cycle. Three causes are identified for its formation. First, the principal one is the decomposition of *N*^*5*^*,N*^*10*^-methylene-tetrahydrofolate (5,10-CH_2_-THF) to form formaldehyde and THF. Increasing the rate of glycine cleavage promotes the formation of 5,10-CH_2_-THF, thereby increasing the formaldehyde release rate. Next, formaldehyde can be produced in the GCS even in the absence of THF. The reason is that T-protein of the GCS can degrade methylamine-loaded H-protein (H_int_) to formaldehyde and ammonia, accompanied with the formation of dihydrolipoyl H-protein (H_red_), but the reaction rate is less than 0.16% of that in the presence of THF. Increasing T-protein concentration can speed up the release rate of formaldehyde by H_int_. Finally, a certain amount of formaldehyde can be formed in the GCS due to oxidative degradation of THF. Based on a formaldehyde-dependent aldolase, we elaborated a glycine-based one carbon metabolic pathway for the biosynthesis of 1,3-propanediol (1,3-PDO) in vitro. This work provides quantitative data and mechanistic understanding of formaldehyde formation in the GCS and a new biosynthetic pathway of 1,3-PDO.

## Introduction

Traditional feedstocks for bioproduction processes mainly base on carbohydrates found in food crops, such as simple sugars and starches. However, the global food shortage caused by the rapid growth of the world’s population and the continuous reduction of available arable land has stunted the development of the bio-manufacturing industry using these feedstocks [1]. Using lignocelluloses as alternative feedstock in biorefinery has an apparent advantage by not competing with human consumption needs, but still faces many technical challenges, [2]. The utilization of one-carbon (C1) compounds, such as CO_2_ [3–5], methane [6], methanol [7] and formate [8], has attracted much attention because they are naturally abundant, or available as industrial by-products and are thus cheap for the production of high-value chemicals. Beyond that, the biological assimilation of C1 compounds for the production of value-added chemicals can help reduce the emission of greenhouse gases such as CO_2_ and methane which are largely responsible for global warming and climate change [4]. In nature, C1 assimilation pathway exists in most methylotrophic bacteria [9]. Upon entrance into bacterial cells, almost all C1 compounds such as methanol, methane, formate and dichloromethane are first oxidized or reduced to formaldehyde and subsequently assimilated into the central part of the metabolism of methylotrophic bacteria [10]. Formaldehyde can be further metabolized either via the serine pathway, the ribulose monophosphate (RuMP) pathway or the Calvin-Benson-Bassham (CBB) cycle in native methylotrophs [11]. Although methylotrophic bacteria can assimilate C1 compounds, their low specific growth rate is less suitable for large industrial production, and the improvement of natural C1 metabolic pathways in native hosts is often difficult due to lack of efficient genetic tools [12]. Hence, it is desirable to introduce synthetic C1 assimilation pathways to model microorganisms such as *Saccharomyces cerevisiae* and *Escherichia coli*, which have been extensively exploited in the biotechnology industry [13]. Recent studies have demonstrated that a wide variety of non-native metabolic pathways can be integrated into these model microorganisms, allowing the synthesis of value-added chemicals, such as 1,3-propanediol (1,3-PDO). At present, the major 1,3-PDO biosynthesis pathways include the routes “glycerol to 1,3-PDO”, “glucose to 1,3-PDO via glycerol” and “glucose to 1,3-PDO via homoserine” [14]. Our recent study [15] showed a novel pyruvate-based C1 metabolic pathway to synthesize 1,3-PDO from formaldehyde and glucose. It was successfully implemented in *E. coli* and demonstrated that C1 compounds like methanol can be used to synthesize 1,3-PDO via formaldehyde as a metabolic intermediate. The incorporation of C1 compounds into the formation of 1,3-PDO synthesis opens up the possibility of incorporating CO_2_ into the production of bulk-chemicals like 1,3-PDO since formaldehyde and methanol can be generated from CO_2_ electrochemically. However, formaldehyde as a substrate suffers from the problem of toxicity to cell growth. Although methanol is less toxic to most microorganisms, the reaction catalyzed by methanol dehydrogenase is thermodynamic unfavorable and limits the conversion rate of this pathway. One of the purposes of the present work is to explore the possibility of generating formaldehyde from glycine using the glycine cleavage system (GCS) for 1,3-PDO synthesis. This may help to circumvent critical issues like substrate toxicity and limited conversion rate in the direct use of formaldehyde and/or methanol. GCS is widely present in the mitochondria of plant and animal tissues as well as in the cytosol of most bacteria. It consists of four different component proteins named as P-protein (glycine decarboxylase; EC 1.4.4.2), T-protein (aminomethyltransferase; EC 2.1.2.10), L-protein (dihydrolipoyl dehydrogenase; EC 1.8.1.4) and H-protein (lipoamide-containing aminomethylene carrier) and catalyzes the oxidative decarboxylation and deamination of glycine to yield one molecule each of CO_2_ and ammonia, in accompany with the transfer of a methylene group to tetrahydrofolate (THF), forming thereby 5,10-CH_2_-THF (Eq. 1):

1 $$ \mathrm{Glycine}+{\mathrm{NAD}}^{+}+\mathrm{THF}\leftrightarrow {\mathrm{CO}}_2+{\mathrm{NH}}_3+5,10-{\mathrm{CH}}_2-\mathrm{THF}+\mathrm{NADH}+{\mathrm{H}}^{+} $$

**Fig. 1:**
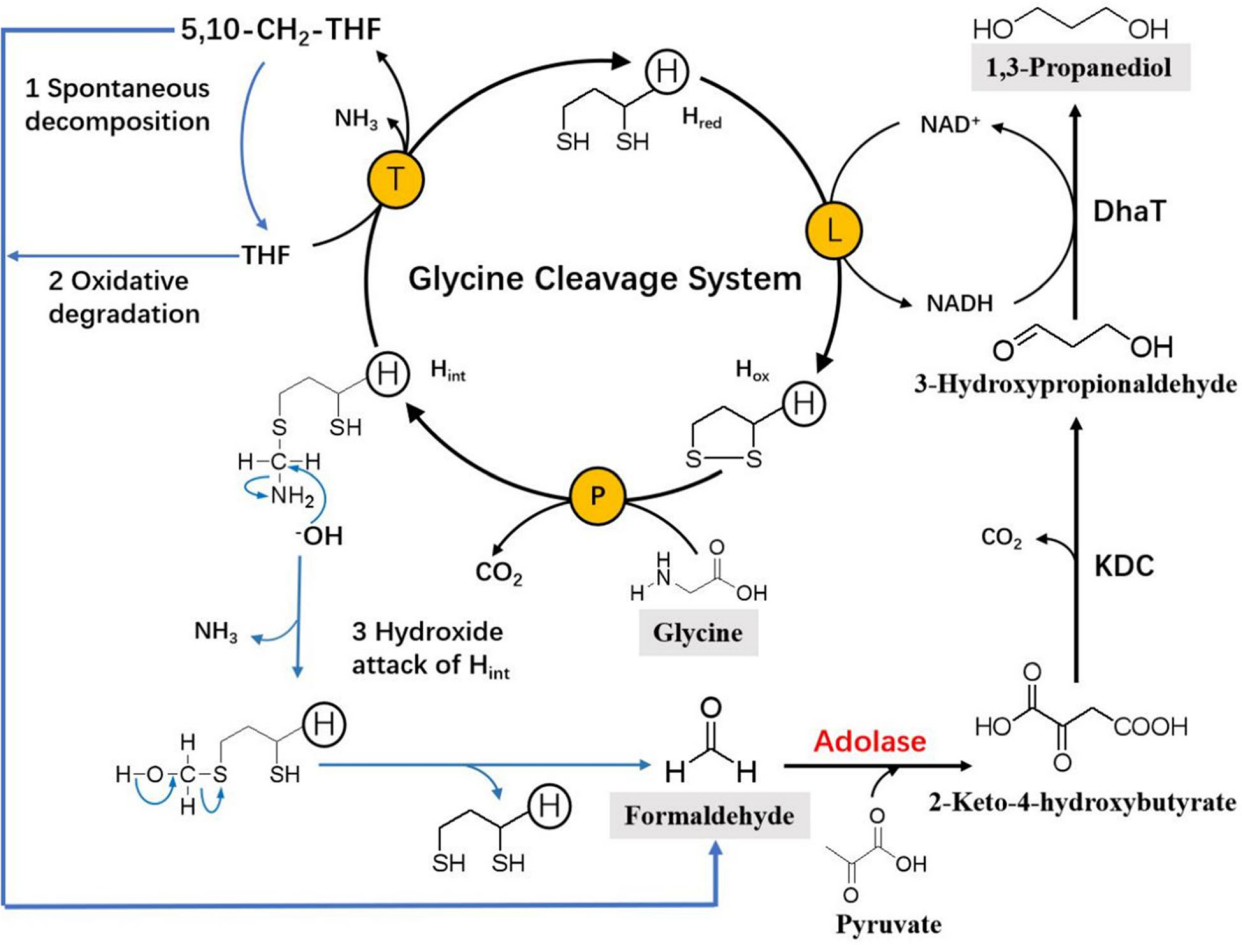
Schematic presentation of the glycine cleavage system (GCS), the possible routes of formaldehyde formation and the direct use of generated formaldehyde in a novel synthesis pathway of 1,3-propanediol based on a pyruvate-dependent aldolase

In the enzymatic reaction cycle of the GCS (Fig. 1), lipoylated H-protein plays a pivotal role by acting as a mobile substrate, which interacts with the three other proteins via its freely swinging lipoyl arm. The degradation of glycine molecules is first triggered by P-protein to yield CO_2_ and methylamine-loaded H-protein (H_int_). H_int_ then forms a complex with T-protein, leading to a degradation of the aminomethyl moiety to ammonia and 5,10-CH_2_-THF in the presence of THF, whereas H_int_ leaves as dihydrolipoyl H-protein (H_red_) in a reduced form. After that H_red_ is oxidized to the oxidized form of H-protein (H_ox_) under the catalysis of the L-protein and the accompanying conversion of NAD^+^ to NADH [16]. The formation of NADH is usually used for assay of the GCS reaction rate.

Kikuchi et al. [17] discovered formaldehyde formation in the GCS. Guilhaudis et al. [18] reported the decomposition of H_int_ to H_red_ and formaldehyde in the absence of THF. However, till now, no systematic studies have been carried out on the formation of formaldehyde in the GCS and the reasons for its formation are not yet clear. In this study, we carried out a systematic and quantitative study and first revealed three possible sources of formaldehyde formation in the GCS. Furthermore, we demonstrated that formaldehyde generated from glycine via the GCS is able to be directly used for the synthesis of 1,3-PDO through our recently proposed aldolase-based metabolic pathway [15]. It is noted that although glycine is a C2 compound used here for 1,3-PDO synthesis, the study of this new pathway could give some useful hints for the utilization of C1 compounds such as formate. Similar to the glycine utilization via GCS to generate 5,10-CH2-THF (Fig. 1), formate can be catalyzed by formate tetrahydrofolate ligase and the bifunctional methenyltetrahydrofolate cyclohydrolase/ dehydrogenase to generate 5,10-CH2-THF which is decomposed to formaldehyde and thus also used for the synthesis of 1,3-PDO.

## Materials and methods

### Materials

All chemicals were of reagent grade and purchased from commercial suppliers including Aladdin (Shanghai, China), Yuanye Bio-Technology (Shanghai, China), Sigma-Aldrich (St. Louis, MO, USA), unless otherwise noted. Phusion High-Fidelity DNA polymerase and restriction enzymes were purchased from Thermo-Fisher Scientific (Pittsburgh, PA, USA). T4 DNA ligase was purchased from New England Biolabs (Ipswich, MA, USA). DNA extraction kit and gel extraction kit were purchased from Promega (Madison, WI, USA). Oligonucleotide primers were ordered from GeneWiz (Suzhou, China). Ni^2+^-NTA resin was purchased from Genscript (Nanjing, China). Amicao® Ultra-15 filtration devices (molecular cutoff 10–100 KDa) were purchased from Millipore (Billerica, MA, USA). BCA protein assay kit and precast-gel for sodium dodecyl sulfate-polyacrylamide gel electrophoresis (SDS-PAGE) were purchase from Solar Bio (Beijing, China).

### Plasmid construction, protein expression and purification

Genes encoding P-protein, T-protein, H-protein, L-protein and SHMT (serine hydroxymethyltransferase) were amplified from the genomic DNA of *E. coli* K12 and cloned into corresponding expression vectors (Table 1). *E. coli* BL21 cells were used as the host for protein overexpression and purification. The recombinant strains harboring pET-P, pET-T, pET-L, pET-H and pET-SHMT, respectively, were grown at 37 °C in LB-medium supplemented with an appropriate antibiotic (100 μg/mL ampicillin or 50 μg/mL kanamycin) until the OD_600_ reached 0.8, and gene expression was then induced by adding 0.2 mM IPTG for additional 12 h at 30 °C. From each culture, cells were collected by centrifugation at 3500 rpm for 10 min, resuspended in 50 mM Tris-HCl (pH 7.5), and disrupted by sonication. Cell debris was removed by centrifugation at 100,000 rpm for 1 h, and the supernatant was purified using a Ni^2+^-NTA column to obtain purified enzymes for activity assays. Purified proteins were checked by SDS-PAGE and protein concentrations were quantified using BCA protein assay kit.

**Table 1:** Strains and plasmids used for this study

	Description	Reference
***E. coli*** **Strains**		
Top 10	Host for cloning plasmids	WEIDI Ltd.
BL21 (DE3)	Host for protein overexpression and purification	WEIDI Ltd.
**Plasmids**		
pET28a (+)	Plasmid for protein overexpression	Novagen
pET22b (+)	Plasmid for protein overexpression	Novagen
pET28a-P	pET28a vector containing P-protein gene (NCBI No. WP_112929453.1)	This study
pET28a-T	pET28a vector containing T-protein gene (NCBI No. WP_099356926.1)	This study
pET28a-H	pET28a vector containing H-protein gene (NCBI No. WP_001295377.1)	This study
pET28a-L	pET28a vector containing L-protein gene (NCBI No. WP_110826218.1)	This study
pET22b-SHMT	pET22b vector containing SHMT gene (NCBI No. WP_072756891.1)	This study
pRSFduet-1-KHB-KDC-DhaT	pRSFduet-1 vector containing KHG gene (NCBI No. WP_000800512), KDC gene (NCBI No. WP_046124870) and DhaT gene (NCBI No. WP_004900746)	Laboratory stock [15]

### Glycine cleavage system activity assay

The standard reaction mixture contained Tris-HCl (50 mM, pH 7.5), 0.5 mM THF, 20 mM β-mercaptoethanol (MCE), 25 μM pyridoxal 5′-phosphate (PLP), 5 mM NAD^+^, 5 μM P-protein, 5 μM T-protein, 5 μM L-protein and 10 μM H-protein. After premixing and centrifugation, reactions were initiated by the addition of 50 mM glycine. Glycine degradation was determined at 37 °C by measuring NADH formation using an Enspire multimode plate reader (PerkinElmer, USA) as described previously [19, 20].

### Inhibitory effect of formaldehyde on the activity of the GCS

The investigation of formaldehyde toxicity was performed by adding various concentrations of formaldehyde to the GCS reaction mixture to determine the initial rates of the GCS. The composition of the standard reaction mixture was the same as described in 2.3. After premixing and centrifugation, reactions were initiated by the addition of 50 mM glycine and different concentration of formaldehyde (0–5 mM). The initial rates of NADH formation in different concentrations of formaldehyde samples were used to assess the effect of formaldehyde toxicity.

### 1,3-PDO synthesis

The three-enzyme (KHG, KDC, and DhaT) reaction cascade for the synthesis of 1,3-PDO was assayed as described previously [15]. The proof-of-concept biosynthesis of 1,3-PDO was conducted by mixing purified GCS enzymes (5 μM P-protein, 5 μM T-protein, 5 μM L-protein and 10 μM H-protein) and a crude cell extract (containing KHG, KDC, and DhaT) with 50 mM Tris-HCl buffer (pH 7.5), 0.5 mM THF, 20 mM β-mercaptoethanol, 25 μM PLP, 1 mM NAD^+^, 10 mM sodium pyruvate, and 1 mM thiamine pyrophosphate (TPP). The reaction was started by adding 10 mM glycine, kept at 37 °C for 7 h, and analyzed by GC-MS.

### Analytical methods

**Fig. 2:**
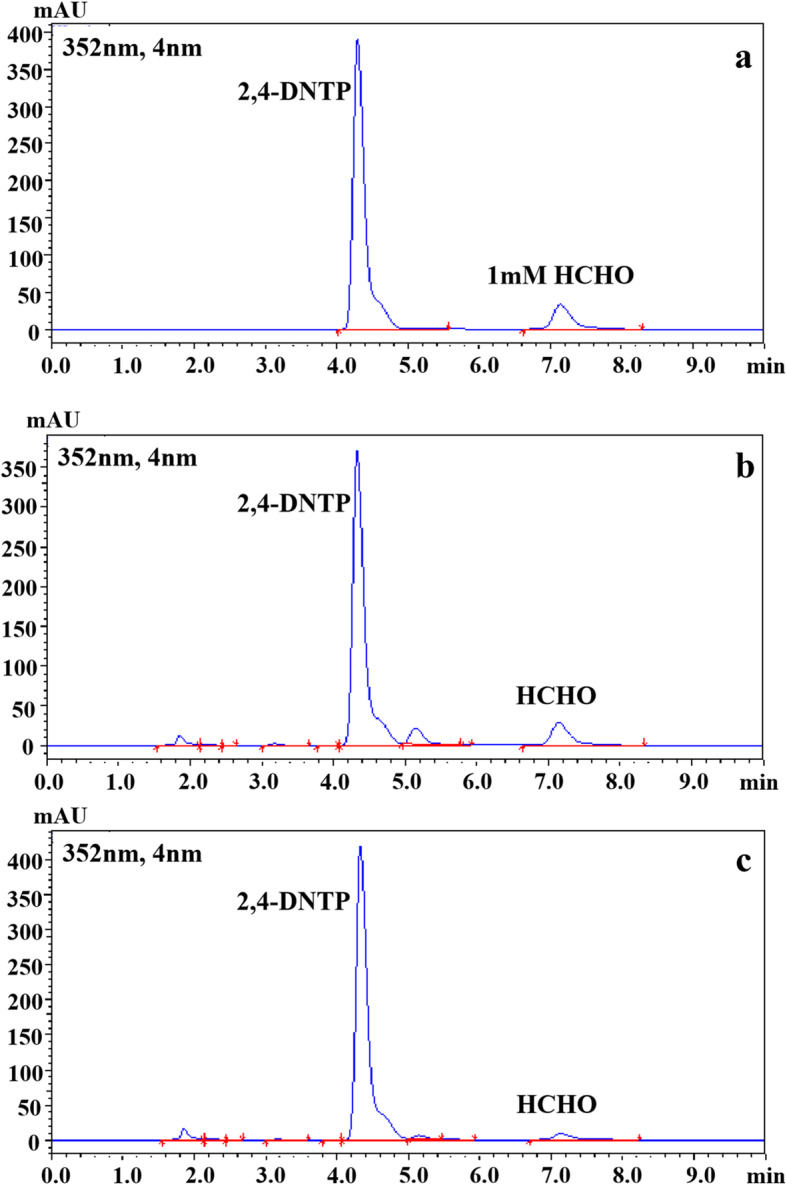
HPLC analysis of formaldehyde from different samples. **a** 1 mM formaldehyde standard. **b** GCS reaction mixture after 2 h. **c** GCS reaction mixture without adding GCS enzymes after 2 h

Free formaldehyde in GCS reaction mixtures was derivatized with 2,4-dinitrophenylhydrazine (2,4-DNPH) to form 2,4-dinitrophenylhydrazone, before quantified by HPLC (Fig. 2). To this end, 0.2 mL of a reaction mixture was mixed with 0.2 mL of 10% (v/v) trifluoroacetic acid (TFA), 0.1 mL of 2,4-DNPH (1 g/L) and 0.5 mL of acetonitrile. Derivatization occurred at 40 °C for 30 min. The 2,4-dinitrophenylhydrazone derivative was analyzed using a Wondasil C_18_ column (4.6 mm × 150 mm, 5 μm, Shimadzu, Japan) with acetonitrile/water (50,50) containing 0.095% TFA as the mobile phase at a flow rate of 1.0 mL/min, and detected at the wavelength of 352 nm.

1,3-PDO was analyzed by GC-MS as described in Wang et al. using a QP2020 system (Shimadzu, Japan) equipped with a SH-Rxi-5Sil-MS column (Shimadzu, Japan), with helium as the carrier gas. The oven temperature was programmed to be held at 100 °C for 2 min, raised at a gradient of 15 °C min^− 1^ to 270 °C and held for 12 min at 270 °C.

## Results and discussion

### Formaldehyde as a by-product in the GCS reaction

**Fig. 3:**
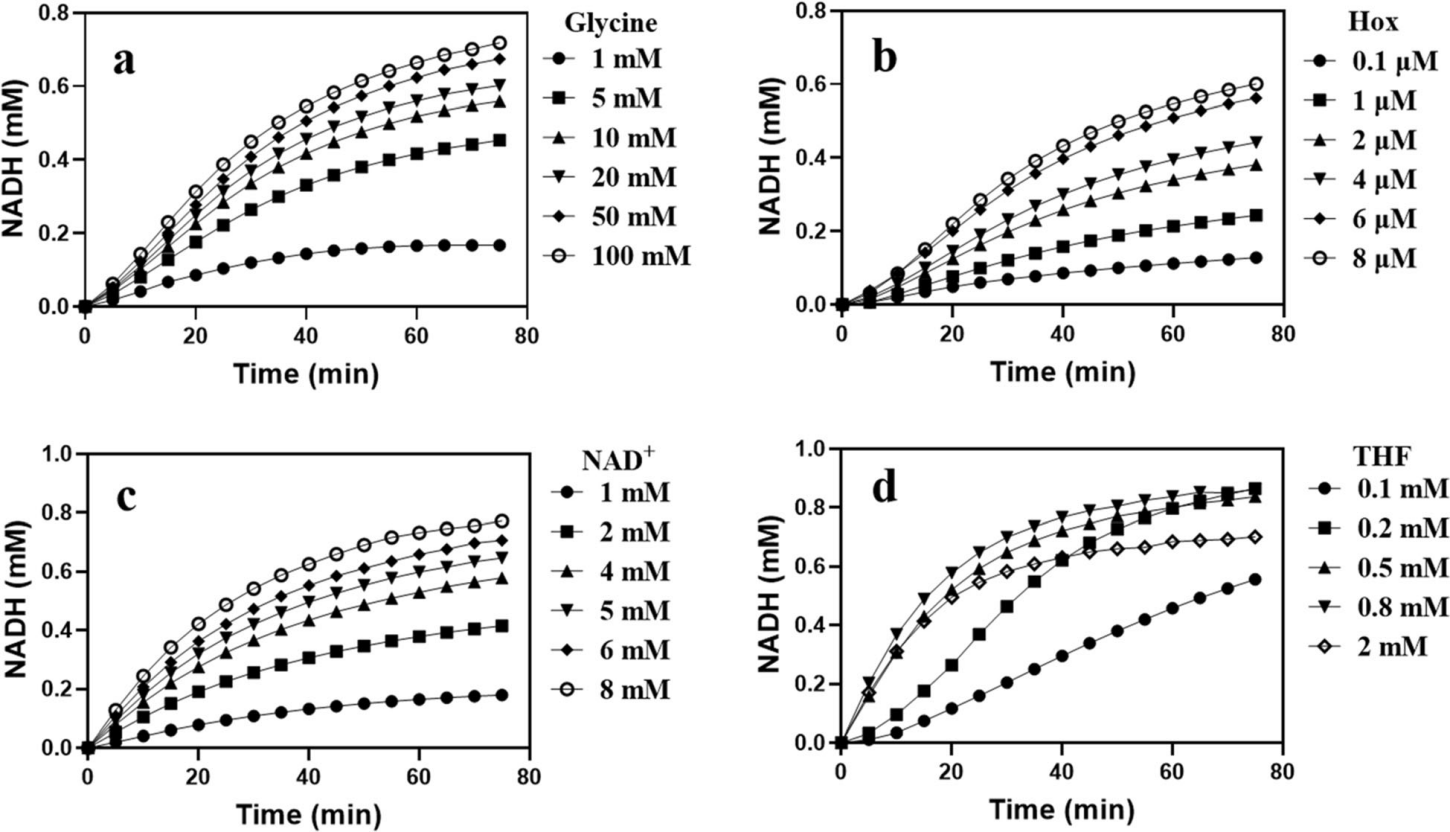
Effects of concentrations of different substrates on the overall GCS reaction rate as determined by the time course of NADH production in the presence of **a** and **b** 0.2 μM P-protein, **c** 0.2 μM L-protein and **d** 0.2 μM T-protein. THF in **a**, **b** and **c** was 0.5 mM. Other reaction components and GCS enzymes are present in excess as specified in Materials and Methods

In our initial study of the GCS as a potentially important route for formate-based biosynthesis, a series of experiments at different concentrations of glycine, H_ox_, NAD^+^ and THF were carried out and the NADH production was monitored as a measure of the reaction rate of the GCS. As shown in Fig. 3, while increasing the concentration of glycine, H_ox_ or NAD^+^ the initial reaction rate increased gradually, but in the case of THF, the reaction first showed a strong acceleration when the concentration of THF added increased from 0.1 to 0.5 mM, and then stagnation when the concentration of THF was further raised to 0.8 mM, and even decrease when 2 mM THF was used. According to a previous study [21], high concentrations of THF can inhibit the conversion of glycine to CO_2_ catalyzed by P-protein, which may be the reason for the results shown in Fig. 3d. According to Eq. 1 the formation of NADH is stoichiometrically linked to the consumption of THF and the corresponding formation of 5,10-CH_2_-THF from the methylene carbon unit of glycine and THF. Unexpectedly, the production of NADH exceeded the added amount of THF (0.5 mM) in Figs. 3a-c, when the initially added concentrations of the specific substrates in Figs. 3a-c increased above certain levels. This was also observed in Fig. 3d except for the case with 2 mM THF, where a reaction inhibition by THF was obvious. For the stoichiometric discrepancy between the formation of NADH and the consumption of THF a possible explanation would be that 5,10-CH_2_-THF formed in the GCS reaction is highly instable and can be spontaneously decomposed into formaldehyde and THF under the given experimental conditions (pH 7.5) [22]. THF then enters again into the GCS reaction, leading to the further conversion of glycine and the formation of additional NADH.

To verify the supposition, an assay method to determine free formaldehyde using HPLC was established, with a pre-column derivation of formaldehyde with 2,4-dinitrophenylhydrazine. The results of HPLC (Fig. 2) showed that formaldehyde was indeed formed in the reaction samples. Thus, it is proved that formaldehyde is one product of the in vitro GCS reaction in this study.

**Fig. 4:**
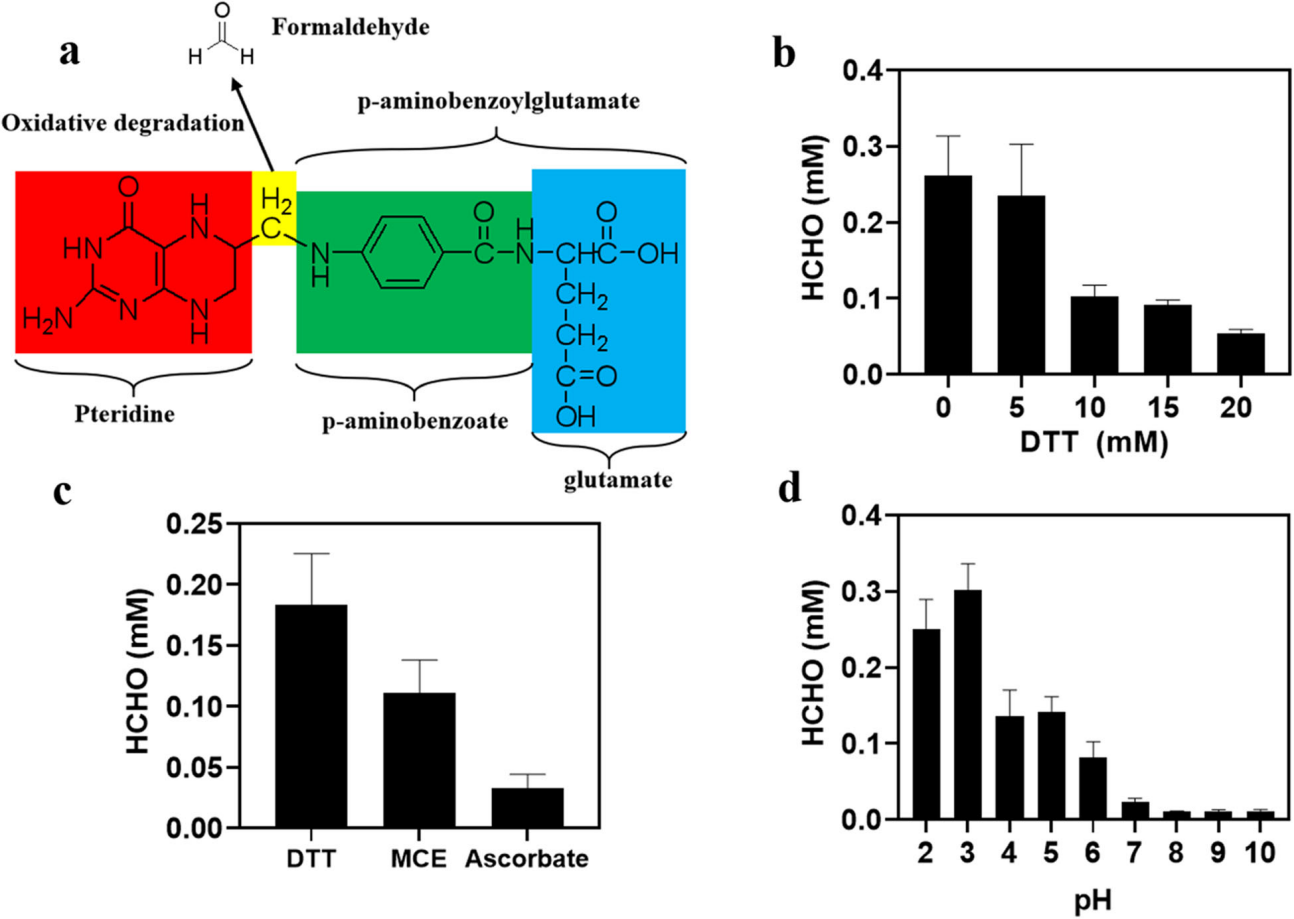
Release of formaldehyde by the degradation of THF. **a** Schematic diagram of formaldehyde released from THF molecule. **b** Effect of DTT concentration on formaldehyde released from THF. The reaction mixture contained 0.5 mM THF, 50 mM Tris-HCl buffer (pH 7.5) and different concentration of DTT, and was assayed after 6 h. **c** Effect of different antioxidants on the release of formaldehyde from THF degradation. The reaction mixture contained 0.5 mM THF, 50 mM Tris-HCl buffer (pH 7.5) and 2 mM antioxidant after 3 h. **d** Formaldehyde released from THF at different pH values. The reaction mixture contained 0.5 mM THF, 20 mM DTT in phosphate buffer at different pH values

It should be mentioned that formaldehyde was also detected in the reaction mixture free of GCS enzymes after 2 h, as shown in Fig. 2c. To find out the reason for the existence of formaldehyde in the enzyme-free reaction mixture, we determined the formaldehyde concentrations in solutions of the different substrates of the GCS reaction mixture, respectively. The results revealed the presence of a small amount of formaldehyde only in the THF solution (0.06 mM formaldehyde in 0.5 mM THF solution). As indicated in literature [23], THF decomposition through oxidative degradation may release formaldehyde as well (Fig. 4a). To systematically examine THF stability with regard to formaldehyde formation, THF solutions containing different concentrations of dithiothreitol (DTT) as antioxidant was placed in the air at 37 °C. Figure 4b shows that exposure to air resulted in the oxidative decomposition of THF to formaldehyde, but the amount of formaldehyde released from THF decreased with increased concentration of DTT. Comparing three commonly used antioxidants, ascorbate has the strongest ability to prevent the release of formaldehyde by THF degradation, followed by MCE and DTT (Fig. 4c). The result in Fig. 4d demonstrates that regarding formaldehyde formation THF was also susceptible to acidic conditions and the release of formaldehyde increased with increased acidity.

### Factors affecting formaldehyde formation in the GCS reaction

**Fig. 5:**
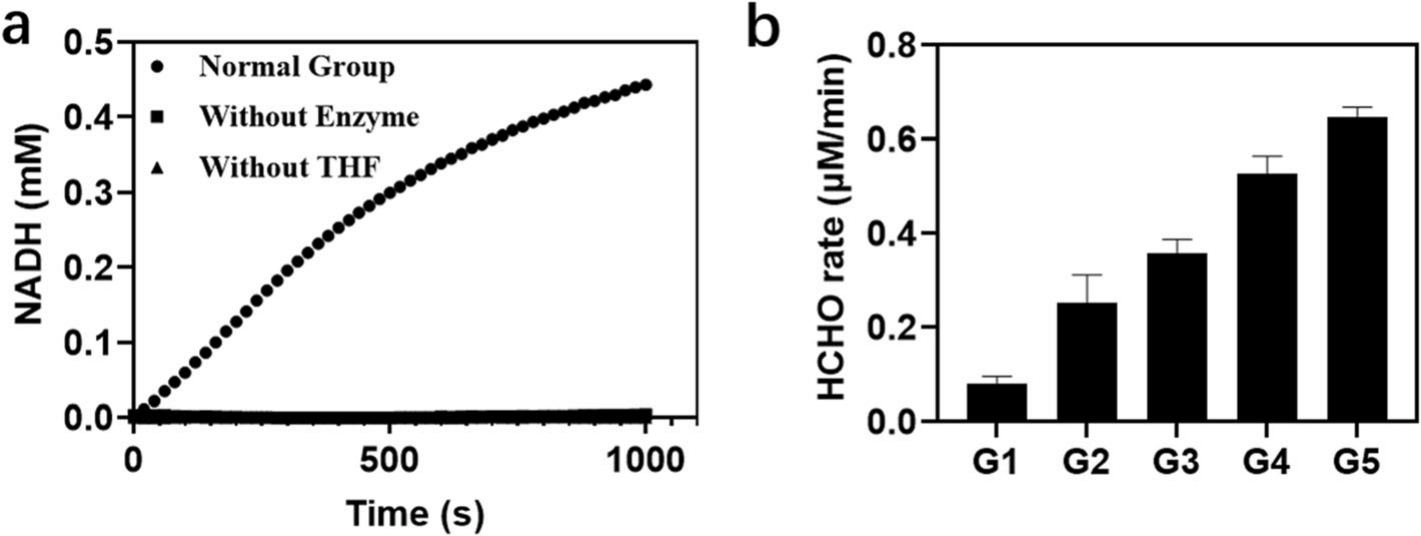
**a** GCS activity assay confirming the necessity of THF for the operation of the whole system. “Normal Group” refers to a GCS reaction mixture as specified in the Materials and Methods section without missing any reaction components and enzymes. “Without Enzyme” refers to a reaction mixture containing the same concentrations of substrates except that no GCS enzymes were added. “Without THF” refers to a reaction mixture containing all reaction components and enzymes except for THF. **b** Formaldehyde formation in the overall GCS reaction catalyzed by T-protein at concentrations of 5, 10, 25, 50, and 80 μM from G1 to G5, respectively, after 5 h. Other reaction components and enzymes were the same as described in the Materials and Methods section

We sought to assess the effects of key factors affecting the GCS reaction activity and formaldehyde formation. Similar to the control group without adding GCS enzymes (without enzyme) and in contrast to the normal group, there was almost no NADH formation in the experimental group without adding THF (without THF) (Fig. 5a). Therefore, THF plays an indispensable role in initiating and accelerating the GCS reaction rate. Interestingly, in the absence of THF, T-protein was still able to cause a change in the overall conformation of the H_int_ (methylaminated form), leading to the release of the lipoamide methylamine arm from the cleft at the surface of the H-protein. According to Guilhaudis et al. [18] this situation favors nucleophilic attack by OH^−^ of the carbon atom in the aminomethyl group. Such an attack can lead to a slow release of NH_3_ and formaldehyde, which is accompanied by the full reduction of the lipoamide arm (Fig. 1). As shown in Fig. 5b, T-protein catalyzed the release of ammonia and formaldehyde from H_int_ in the absence of THF, and the reaction rate increased with increased concentration of T-protein. However, at the same concentration of T-protein (5 μM), the reaction rate of T-protein catalyzed release of formaldehyde (0.08 μM/min) was less than 0.16% of the formaldehyde formation rate (50 μM/min) measured in the presence of THF. Thus, in the absence of THF, the methylamine transfer reaction rate catalyzed by T-protein is extremely low, which is not the primary cause of formaldehyde formation in the GCS reaction system. A similar suggestion was given by Kikuchi et al. [17] but without providing any quantitative data.

**Fig. 6:**
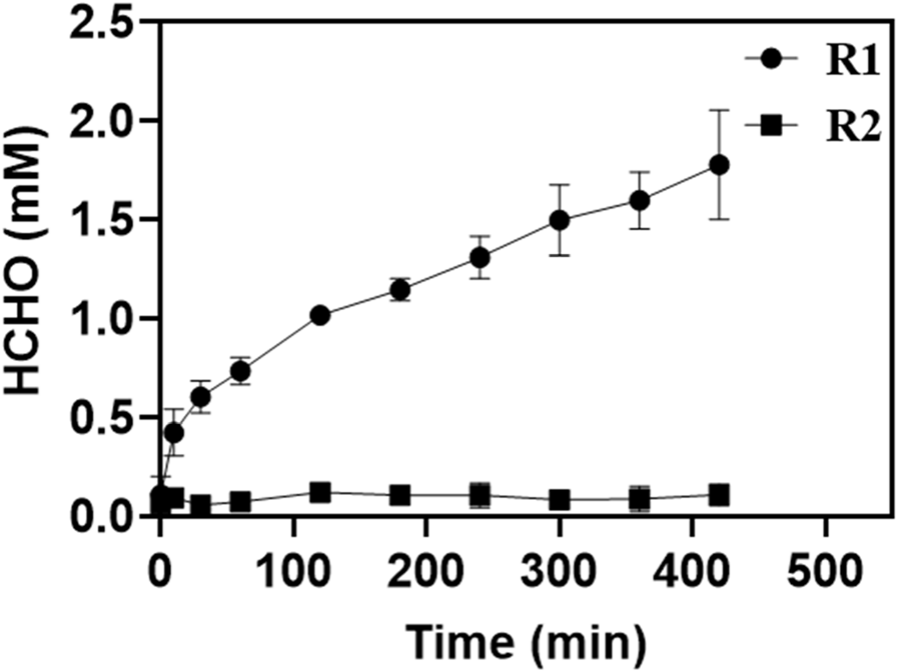
Effect of SHMT on formaldehyde production in the GCS reaction. R1 indicates a GCS reaction mixture as specified in the Materials and Methods section. R2 refers to a reaction mixture similar to R1 with the addition of SHMT

Margaretha et al [22] reported that 5,10-CH_2_-THF dissociates into THF and formaldehyde at physiological pH and acidic pH. In fact, 5,10-CH_2_-THF is only stable towards dissociation into THF at pH > 8 or in the presence of a huge molar excess of formaldehyde. Since the GCS reaction mixture used in the present work was buffered at pH 7.5, 5,10-CH_2_-THF was more easily decomposed into formaldehyde and THF. The generated THF re-entered the reaction so that the overall GCS reaction proceeded to an extent, leading to higher NADH production than stoichiometrically expected. In order to further prove our conjecture, we constructed in vitro a GCS-SHMT cascade reaction system to timely remove the GCS-catalyzed product 5,10-CH2-THF. Serine hydroxymethyltransferase (SHMT; EC 2.1.2.1) is a pyridoxal 5′-phosphate (PLP) dependent enzyme that catalyzes the reversible conversion of 5,10-CH_2_-THF and glycine to THF and serine and therefore closely associated with the function of the GCS in C1 metabolic pathways. The results in Fig. 6 show that compared with the continuous increase in the concentration of formaldehyde in the R1 group (containing only GCS enzymes) over time, the concentration of formaldehyde in the R2 group (containing SHMT in addition to the GCS enzymes) did not increase with time and remained at almost zero, indicating that most formaldehyde formed in the GCS reaction was from decomposition of 5,10-CH_2_-THF.

**Fig. 7:**
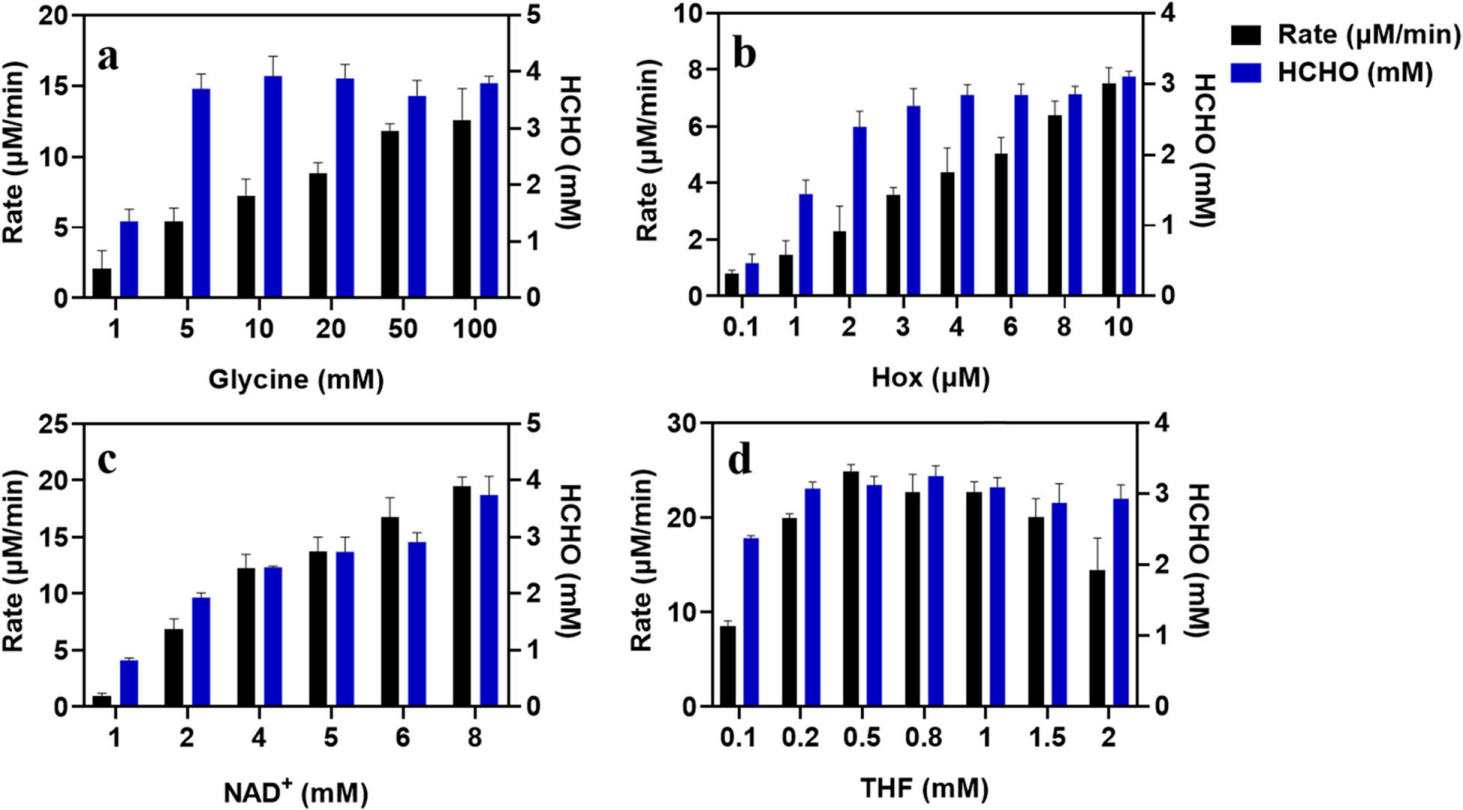
Effects of concentrations of different substrates on the formation rate and final concentration of formaldehyde in the GCS reaction. The compositions of the reaction mixtures were as specified in the Materials and Methods section, except for that in **a** and **b** P-protein added was 0.2 μM, in **c** T-protein added was 0.2 μM, and in **d** L-protein added was 0.2 μM

We further observed that concentrations of substrates not only affected the overall reaction rate of the GCS (Fig. 3), but also determined the rate of formaldehyde formation, as shown in Fig. 7. The overall reaction rate of GCS increased gradually as the substrate concentration (either glycine, H_ox_, or NAD^+^) was increased (Fig. 3), and increasing the rate of glycine cleavage promoted the formation of 5,10-CH_2_-THF, thereby increasing the formaldehyde release rate. In the case of THF increased concentration led first to higher formaldehyde generation rate, however, further increase in the concentration of THF resulted in decrease in the rate of formaldehyde formation. This can be explained by the inhibition behavior of a high concentration of THF both on the overall GCS reaction and on the dissociation of 5,10-CH_2_-THF (Fig. 3d). To better understand the relationship between the concentration of THF and the rate of formaldehyde formation, the construction of a GCS kinetic model is desirable.

Concentrations of substrates added into the reaction mixture also affected the final yield of formaldehyde. According to the experimental results in Fig. 7, when the initial concentration of a substrate (either glycine, H_ox_, or NAD^+^) was increased, the final concentration of formaldehyde generated increased as well. However, when the initial substrate concentration was above a certain level, the final concentration of formaldehyde reached did not further increase with increased substrate concentration but remained nearly constant. This may be due to the toxicity of formaldehyde which is significant at a concentration above 3–4 mM (data not shown). In the case of THF as shown in Fig. 7d, even at relatively low THF concentrations, the formation of formaldehyde in the GCS reaction was already significant.

### 1,3-PDO formation from formaldehyde via glycine

**Fig. 8:**
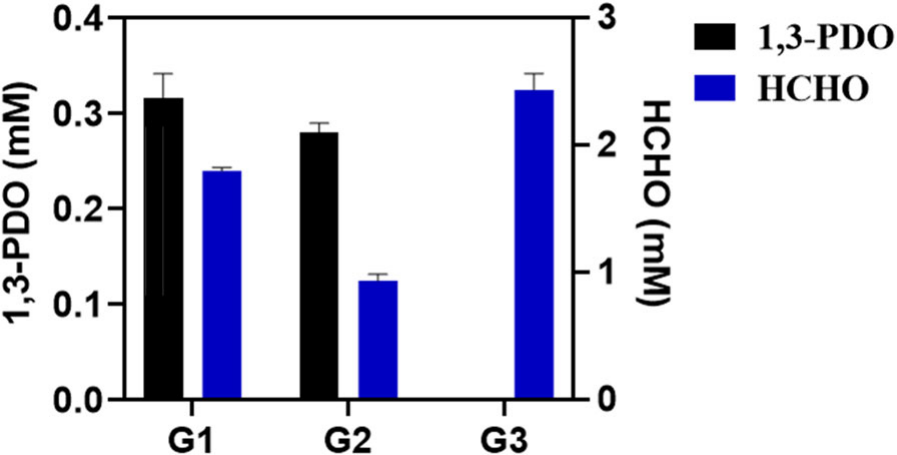
In vitro 1,3-PDO synthesis. G1: glycine and pyruvate as substrates via GCS coupled with the enzymes KHG, KDC, and DhaT; G2: formaldehyde (5 mM) and pyruvate as substrates with only the three enzymes KHG, KDC, and DhaT; G3: glycine as substrate catalyzed by GCS without adding KHG, KDC, and DhaT

In a previous work of our research group, we successfully used a pyruvate-dependent aldolase to condense formaldehyde and pyruvate into 2-keto-4-hydroxybutyrate (HOBA), and utilized two further enzymes, a branched-chain alpha-keto acid decarboxylase (KDC) and a NADH-dependent 1,3-PDO oxidoreductase (DhaT), to convert HOBA into 1,3-PDO. In the present work, instead of directly using formaldehyde as a substrate, we utilized glycine as a starting material and formaldehyde dissociated from 5,10-CH_2_-THF, which is generated in the GCS reaction cycle, to synthesize 1,3-PDO (Fig. 1). As shown in Fig. 8, 1,3-PDO was indeed in vitro synthesized in such an enzyme cascade reaction system with glycine and pyruvate as substrates. Slightly higher 1,3-PDO concentration was achieved in comparison with the direct use of formaldehyde and pyruvate. The use of glycine has the following advantages: (1) reduced toxicity towards the enzymes used compared to the direct use of formaldehyde; and (2) NADH produced by the GCS can be utilized for the reduction of 3-HPA to 1,3-PDO catalyzed by DhaT. It is known that the yield of 1,3-PDO is mainly limited by the availability of reducing power in form of NADH or NADPH.

It should be mentioned that the possibility of using formaldehyde from the GCS reaction cycle for in vivo 1,3-PDO biosynthesis should be experimentally checked carefully, since 5,10-CH_2_-THF is involved in many bioreactions in vivo [24]. Furthermore, the intracellular environment conditions should be considered. As mentioned above the existence of SHMT may prevent the accumulation of 5,10-CH_2_-THF and thus the release of formaldehyde. Irrespective of the possibility of using formaldehyde from the GCS reaction cycle for biosynthesis this work provided useful quantitative data and mechanistic understanding of formaldehyde formation in the GCS reaction. Most of previous studies focus on the catalytic mechanism of each components in GCS, and few results were published regarding the kinetic properties of the overall reaction. In fact, a convenient way to better understand the kinetic behavior of GCS and the synergistic effect of each components is to study them as a whole. Compared with previous work, our systematic study on GCS revealed new and quantitative information about glycine-derived formaldehyde formation. Although 5,10-CH_2_-THF is known to dissociate into formaldehyde and THF in vitro, the formation of formaldehyde in GCS under the presence of THF is first quantitatively studied in this work. We also confirmed the significance of THF in promoting the overall GCS reaction, and studied the factors affection the oxidative degradation of THF to release formaldehyde. Importantly, the results clearly showed that the measurement of NADH is not a reliable method to study the kinetics of GCS reactions since its formation rate is affected by the availability of THF. The latter is involved in two of the three mechanisms of formaldehyde formation related to the GCS system which were examined in this work. As recently pointed out by Hong et al [24] it is desired to develop more efficient and reliable analytic methods for direct array of the individual reaction steps of the GCS system.

## Conclusion

In this work, we studied the kinetic behavior of the GCS and proved that a substantial amount of formaldehyde was formed in the GCS reaction cycle. Three sources of formaldehyde formation were identified. It was shown that the main reason of formaldehyde formation was caused by the dissociation of 5,10-CH_2_-THF under the given experimental conditions. The formation of formaldehyde in GCS should be carefully considered in studying GCS and using it for C1-based synthetic biology. Combining the findings from this work with previous research results of our lab, we constructed a novel 1,3-PDO biosynthetic pathway with glycine and pyruvate as substrates, and conceptually proved the feasibility of this pathway in vitro.

## Data Availability

Please contact the corresponding author for data requests.
