# Correction to: Formaldehyde formation in the glycine cleavage system and its use for an aldolase-based biosynthesis of 1,3-propanediol

**DOI:** 10.1186/s13036-020-00250-5

**Published:** 2020-12-07

**Authors:** Yingying Xu, Hao Meng, Jie Ren, An-Ping Zeng

**Affiliations:** 1 Beijing Advanced Innovation Center for Soft Matter Science and Engineering, Beijing University of Chemical Technology, North Third Ring Road 15, Chaoyang District, Beijing, 100029 China; 2 State Key Laboratory for Biology of Plant Diseases and Insect Pests/Key Laboratory of Control of Biological Hazard Factors (Plant Origin) for Agri-product Quality and Safety, Ministry of Agriculture, Institute of Plant Protection, Chinese Academy of Agricultural Sciences, Beijing, 100081 China; 3 Institute of Bioprocess and Biosystems Engineering, Hamburg University of Technology, Denickestrasse 15, 21073 Hamburg, Germany

**Correction to: J Biol Eng 14, 15 (2020)**

**https://doi.org/10.1186/s13036-020-00237-2**

Following the publication of the original article [1], the authors became aware of a typographical error in the title: ‘1,3-propanediol’ was inadvertently spelled as ‘1,3-prodanediol’. This has now been corrected in the title of both the original article as well as this Correction.
